# Grid cells: the missing link in understanding Parkinson’s disease?

**DOI:** 10.3389/fnins.2024.1276714

**Published:** 2024-02-08

**Authors:** Alexander Reinshagen

**Affiliations:** SANA Hospital Leipzig County, Borna, Germany

**Keywords:** grid cell, Parkinson ‘s disease, allocentric, dopamine, medial entorhinal cortex, striatum, striato-HF/EC loop

## Abstract

The mechanisms underlying Parkinson’s disease (PD) are complex and not fully understood, and the box-and-arrow model among other current models present significant challenges. This paper explores the potential role of the allocentric brain and especially its grid cells in several PD motor symptoms, including bradykinesia, kinesia paradoxa, freezing of gait, the bottleneck phenomenon, and their dependency on cueing. It is argued that central hubs, like the locus coeruleus and the pedunculopontine nucleus, often narrowly interpreted in the context of PD, play an equally important role in governing the allocentric brain as the basal ganglia. Consequently, the motor and secondary motor (e.g., spatially related) symptoms of PD linked with dopamine depletion may be more closely tied to erroneous computation by grid cells than to the basal ganglia alone. Because grid cells and their associated central hubs introduce both spatial and temporal information to the brain influencing velocity perception they may cause bradykinesia or hyperkinesia as well. In summary, PD motor symptoms may primarily be an allocentric disturbance resulting from virtual faulty computation by grid cells revealed by dopamine depletion in PD.

## Introduction

“Grid cells are such a beautiful and unique phenomenon in the nervous system that it is tempting to regard them as a crucial element of its design” (Kropff and Treves, 2008).

The existing model of the basal ganglia (BG) and dopamine depletion (DD) in Parkinson’s disease (PD) goes back to the late 1950s (Carlsson et al., 1957; Carlsson and Waldeck, 1958; Ehringer and Hornykiewicz, 1960; Birkmayer and Hornykiewicz, 1961; Hornykiewicz, 1962; Bernheimer and Hornykiewicz, 1965; Carlsson, 1971; Yeragani et al., 2010). This model was further refined in the 1980s and early 1990s with the formulation of the direct and indirect striatal output pathways and the “box-and-arrow” model (Penney and Young, 1986; Albin et al., 1989; DeLong, 1990; Nambu et al., 2002; Plotkin and Goldberg, 2018). Although this “has led to groundbreaking strategies to treat motor disorders” (Plotkin and Goldberg, 2018), it “fails to explain certain clinical findings and leaves a number of paradoxes” (Brown and Marsden, 1998, p. 1801), leaving us “far from a comprehensive mechanistic understanding of the pathophysiology of PD” (Wichmann et al., 2011). Furthermore, neurocomputational models (Gurney et al., 2001a,b; Fiore et al., 2016; Suryanarayana et al., 2019; Hjorth et al., 2020) and also trials with respect to deep brain stimulation (DBS) have demonstrated that current concepts of basal ganglia pathophysiology have reached “the point where total rejection, rather than continual attempts at modification, is necessary” (Montgomery, 2011, p. 14). Numerous critical reviews on this topic underline these limitations (Marsden and Obeso, 1994; Mink, 1996; Nambu, 2008; Wichmann et al., 2011; Plotkin and Goldberg, 2018).

The BG pathway refers to medium spiny neurons (MSN), which form clustered cell groups. These groups are activated not only by active and passive manipulation of one body part, but also by their cutaneous stimulation (Crutcher and DeLong, 1984; Alexander and DeLong, 1985; Carelli and West, 1991; Coffey et al., 2016, 2017) are referred to as single body parts (SBP). These SBP deliver a body-referenced frame that primarily “encodes action *space*” (Klaus et al., 2017) from the egocentric view.

However, as movement is generally *goal-directed,* originating from a starting point and progressing toward an endpoint, and often involving the late-stage positioning of a potential target, it relates to the external environment/space (Redgrave et al., 2010; Penner and Mizumori, 2012; Elliott et al., 2017; Grieves and Jeffery, 2017; Pernia-Andrade et al., 2021). It is therefore essential to consider not only the animal’s perspective of the goal’s position, but also the goal’s representation relative to external contextual features (the allocentric frame; Neely et al., 2008; Chersi and Burgess, 2015).

By applying the allocentric properties of grid cells to parkinsonian symptoms in connection with dopamine depletion (DD), we can gain new insights into the pivotal role of the allocentric brain not just in PD, but also in the inception of movement.

## What we know

### The allocentric space model

**Figure 1:**
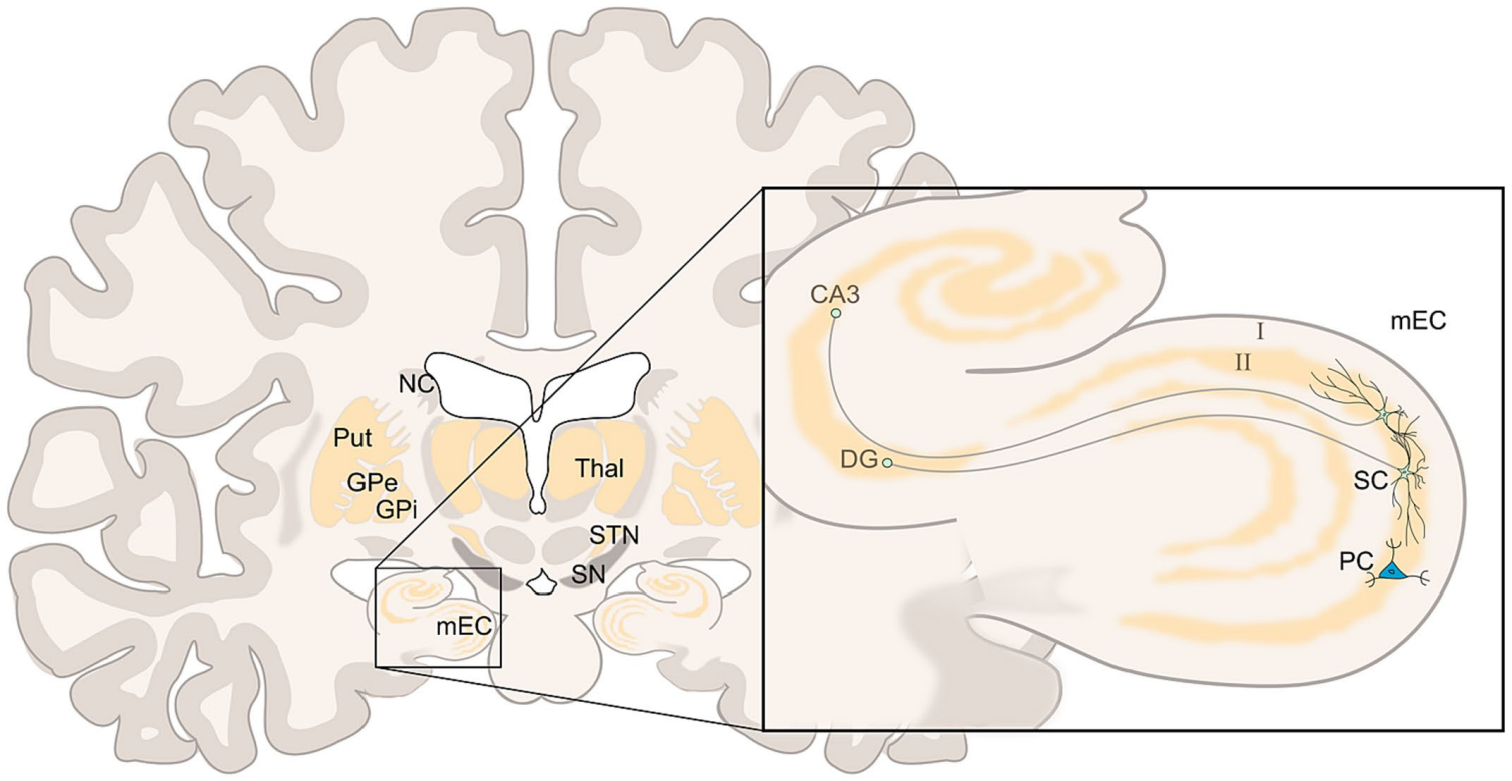
Coronar view of the brain at the EC/HF level (left), in detail (right) two stellate cells (SC), and one pyramid cell (PC) of layer II of mEC, the SC branching within layer II with efferences to the dentate gyrus (DG) and hippocampal cornu ammonis (CA3); left: nucleus caudatus (NC), putamen (Put), and globus pallidus (GPe and GPi) are less voluminous in this level, further shown substantia nigra (SN), subthalamic nucleus (STN), and thalamus (Thal). Adopted from Nieuwenhuys et al. (2008) and Park et al. (2019).

An individual’s objective position and the location of desirable goals are deduced from external landmarks and determined within the hippocampal place cells (O'Keefe and Dostrovsky, 1971). In turn, the computation of allocentric movement, necessary to navigate between landmarks, depends on grid cells (GCs) in the medial entorhinal cortex (mEC)—mainly within layer II in particular (Fyhn et al., 2004; Hafting et al., 2005; Moser et al., 2008; Boccara et al., 2010) (see Figure 1). The mEC consists of about two thirds reelin-positive stellate cells (SC), which supply the dentate gyrus and the hippocampus, also called “ocean cells,” surrounding about one third so-called pyramid cells (PC) or “island cells” projecting to mEC layer I and the contralateral EC. Both SC and PC are influenced by inhibitory microcircuits of different interneurons (Gatome et al., 2010; Witter et al., 2017; Naumann et al., 2018; Tukker et al., 2022) and can function as grid cells with an emphasis on pyramid cells (Tang et al., 2014; Rowland et al., 2018). Grid cells again receive significant dopaminergic innervation (Fallon et al., 1978; Akil and Lewis, 1993; Li et al., 2015).

**Figure 2:**
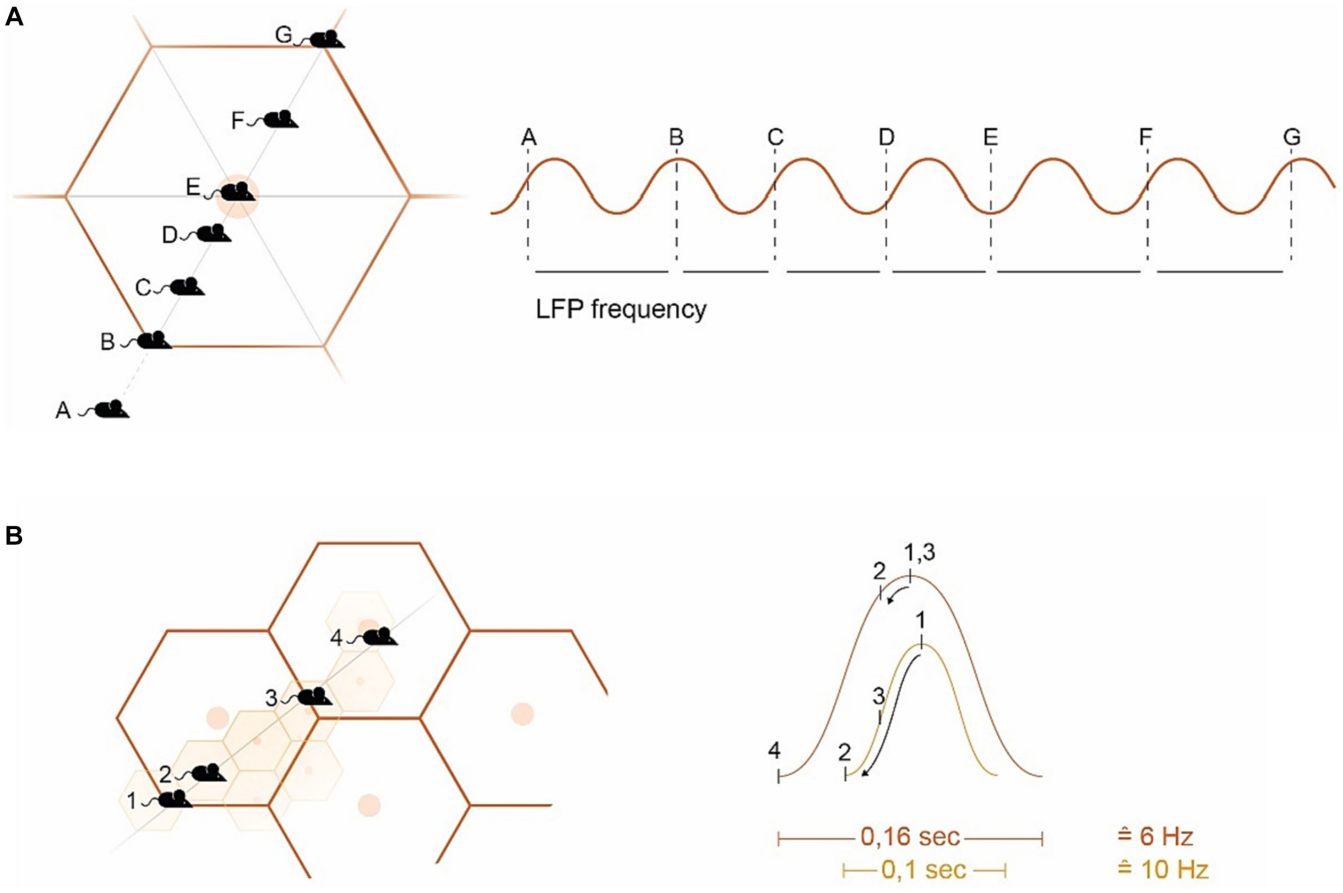
Introducing hexagonal grid fields (GF) **(A)** Example of a mouse navigating a GF. The GC spike timing within the GF aligns with the LFP’s frequency band (sine wave on the right). Starting from the edge of the GF (B), the GC spike occurs at the peak of the SW. As the mouse approaches the GF vertex (C,D), the spiking activity descends on the subsequent SW, ending in the slack portion of the sine curve when the GF vertex is reached (E). Departing from the GF vertex signals conditions climbing up (F) with (G) again starting for the next GF. There is an ambiguity in the directional interpretation of the (A) and (F) signals (toward or away from the vertex) (Mathis et al., 2013). **(B)** Example of overlapping GFs, where large GFs are computed in ventral GC modules at a low frequency (e.g., 6 Hz), and smaller GFs in the dorsal mEC at a higher frequency (e.g., 10 Hz) (simplified representation). Mouse position (MP)1 is at the edge of both the small GF (smGF) and the large GF (lgGF), triggering internal GC spiking at the peak of the sine wave (right). MP2 generates a partial PP from the lgGF, but a substantial PP for the smGF reaching the slack (with the descending arrows within the sine wave indication theta phase precession). MP3 is again on the edge of the lgGF but halfway to the smGF vertex, while MP4 lies on the edge of the smGF but on the vertex of the lgGF. GF, Grid field; smGF, Small GF; lg, Large GF; MP, Mouse position; PP, Phase precession; and SW, Sine wave.

Grid cell generate the external spatial allocentric reference overlaying the animal’s surrounding floor with a two-dimensional hexagonal pattern/carpet (the grid fields) formed by isosceles triangles (Hafting et al., 2005; Burak, 2014; Moser et al., 2014; Knierim, 2015; Vago and Ujfalussy, 2018; Mosheiff and Burak, 2019) (see Figure 2A). The emergence and maintenance of GCs and grid fields rely on exploratory movement, which is anchored to external landmarks and borders (Hafting et al., 2005; Savelli et al., 2008; Couey et al., 2013; Pastoll et al., 2013; Stensola et al., 2015). This movement again generates a self-organizing *internal spiking* within GCs based on local network dynamics (LFP, described in more detail below). For this, similar to striatal SBP, GCs receive spatial information in the form of copies of multimodal sensory input from external landmarks through self-motion data. This data originate from “vestibular, proprioceptive, visual (optic flow) and motor (motor-efference copy) systems” (Jeffery, 2007; Mohedano-Moriano et al., 2008; Barry and Burgess, 2014, p R332; Perez-Escobar et al., 2016; Nadasdy et al., 2017; Campbell et al., 2018), most *visual cues* being particularly relevant (Maaswinkel and Whishaw, 1999; Chen et al., 2013; Kinkhabwala et al., 2020). GCs are grouped into modules that share the same *scale/period* (for *distance* computing) and *orientation* (relative to external references), but have different *phases* (relative positioning of grid fields). These modules present a dorso–ventral gradient within the mEC, with exponentially larger scaling for the ventral modules (Barry et al., 2007; Brun et al., 2008; Stensola et al., 2012; Bant et al., 2020).

### Entorhinal theta phase precession and velocity integration

Within the hippocampal-entorhinal (HF/EC) formation, there is a prominent theta oscillation ranging from approximately 6–11 Hz, known as the “local field potential” (LFP) (Vanderwolf, 1969; Eggink et al., 2014). This oscillation is largely driven by the medial septum (the diagonal band of Broca, MSDB), but underlies the spatial periodicity and internal spiking properties of GCs as well (Burgess et al., 2007; Fyhn et al., 2007; Brandon et al., 2011; Barry et al., 2012; Pilly and Grossberg, 2013; Shay et al., 2016; Jacob et al., 2017; Tsanov, 2017; Joshi and Somogyi, 2020). On the other side active movement stimulates GCs’ internal spiking, which increases in frequency and aligns with the LFP frequency as the vertex of the grid field is approached (Burgess et al., 2007; Giocomo and Hasselmo, 2008; Shay et al., 2016; Gu and Yakel, 2017) such that internal spikes, originating from the peak of the sine wave, descend the wave arriving the slack of the LFP sine *wave* by reaching the vertex of the grid *field*, with the leading spike’s phase delivering mostly spatial information (Reifenstein et al., 2012) (see Figure 2A).

This precession of spikes related to the LFP (i.e., relative to the sum of nearby firing cells) is termed “theta phase precession” (TPP) initially described in place cells (O'Keefe and Recce, 1993; Skaggs et al., 1996) later in GCs as well (Hafting et al., 2008) and again later within the ventral striatum (van der Meer and Redish, 2011; Malhotra et al., 2012). As GCs’ scales compute distances, their TPP signifies distances traveled in a given time that establishes the quality of speed and therefore *time*, *velocity*, and *acceleration* in GC computation (Burgess et al., 2007; Zilli, 2012; Kropff et al., 2021). In this regard, the allocentric computation of GCs introduces the concept of *time* into the brain (Kropff et al., 2015; Heys and Dombeck, 2018; Alexander et al., 2020; Carvalho et al., 2020; Heys et al., 2020; Ridler et al., 2020) (see Figure 2B). This is complemented by the MSDB’s parvalbumin-positive cells modulating the entorhinal LFP’s speed information (Lepperod et al., 2021) and its glutamatergic circuit that controls the initiation and *velocity* of locomotion (Fuhrmann et al., 2015; Justus et al., 2017). I argue that the nature of GC’s theta phase precession (TPP) misgauged in dopamine depletion accounts for bradykinesia generating the slowdown of movement (see Figure 2).

### The link between the striatum and the hippocampal formation

**Figure 3:**
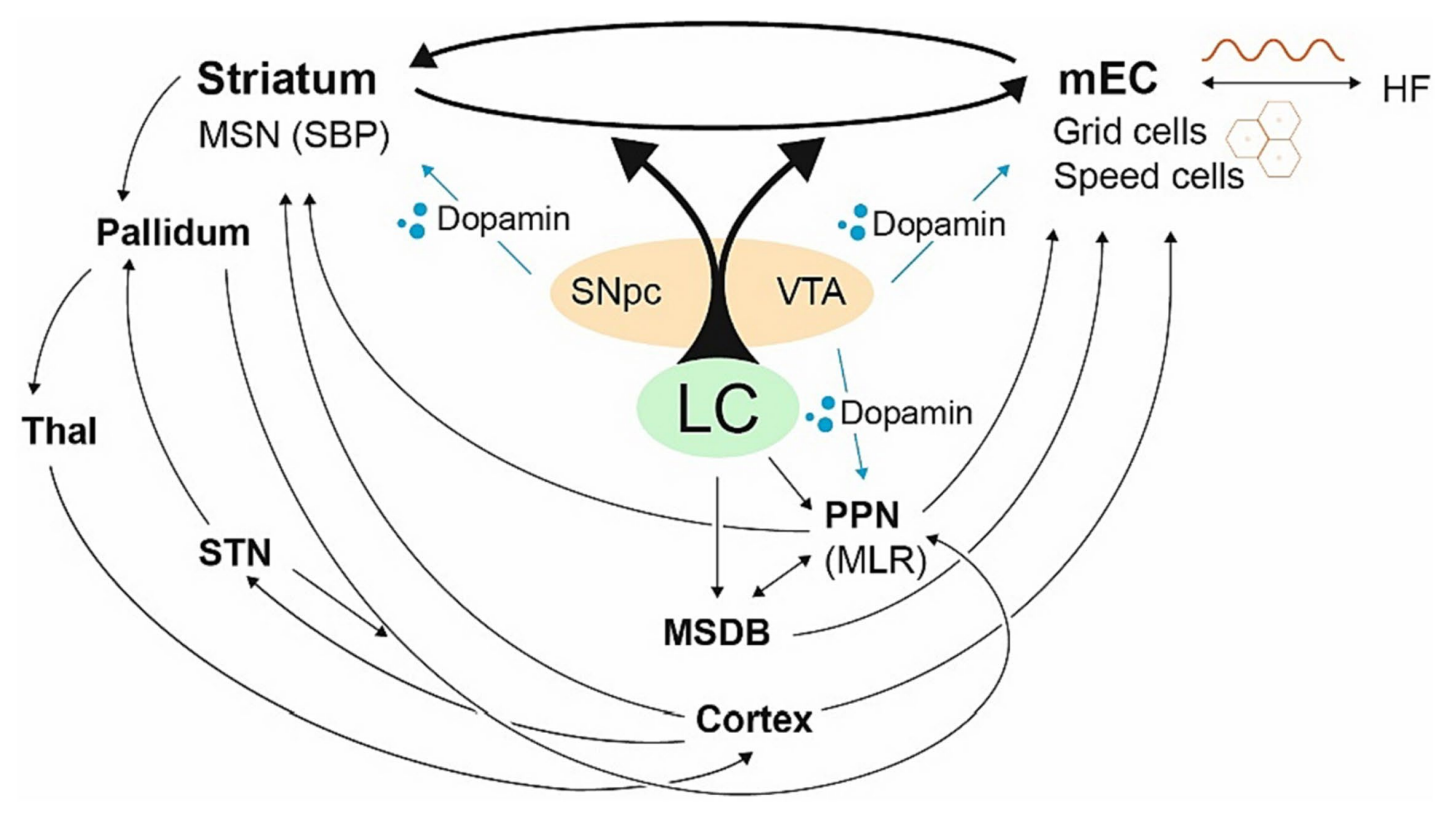
The striato-HF/EC loop with its central hubs. Fragmentary visualization of central hubs dedicated to the basal ganglia and the allocentric brain. The LC balances the striato-HF/EC loop, the HF/EC primarily receives dopaminergic signals from the VTA, the striatum from SNpc. The LC drives the MSDB and the PPN as well driving the HF/EC with grid cells and its LFP (sine wave). The PPN has strong connections with the striatum, modulating the SNpc and VTA (Floresco et al., 2003; Mena-Segovia et al., 2004; Valencia et al., 2014; Roseberry et al., 2016; Mena-Segovia and Bolam, 2017), yet also powerful projections via the MSDB coding mEC speed cells controlling the initiation of locomotion (Fuhrmann et al., 2015). The PPN can be powered by the pallidum and the STN as well. In general, hubs, which are closely related to PD, are heavily involved in the allocentric brain.

To translate goal-directed allocentric components into striatal egocentric self-motion computation and vice versa, the striatum and the HF/EC are effectively linked (Finch et al., 1995; Totterdell and Meredith, 1997; Devan and White, 1999; Hartley et al., 2003; Johnson et al., 2007; Lex and Hauber, 2010; van der Meer et al., 2010; Ghiglieri et al., 2011; Verschure et al., 2014; Stoianov et al., 2018). Constant switching takes place between them (Bohbot et al., 2004; Burgess, 2006; Harris et al., 2012; Colombo et al., 2017), a process that appears to be dopamine-dependent (Penner and Mizumori, 2012) and is driven by the locus coeruleus (LC), the structure to be impaired in PD first (Hornykiewicz and Kish, 1987; German et al., 1992; Braak et al., 2003; Zarow et al., 2003; Braak and Del Tredici, 2017; Vermeiren and De Deyn, 2017; Nahimi et al., 2018; Oertel et al., 2019; Giorgi et al., 2020; Zhou et al., 2021) (see Figure 3). Damage to connecting fibers between the striatum and the HF/EC, the striato-HF/EC loop, disrupts precise navigation in open environments (Devan et al., 1996; Gorny et al., 2002) and the deactivation of one side tends to increase the compensatory use of the other (Packard and McGaugh, 1996; Igloi et al., 2009; Sodums and Bohbot, 2020) with the LC playing a crucial role in creating spatial representations especially sensitive to environmental novelty (Harris et al., 2012; Takeuchi et al., 2016; Ruggiero et al., 2018; Zhong and Moffat, 2018).

For both the striatum and the HF/EC, dopamine is supplied from mesencephalic structures—which undergo loss of dopaminergic neurons in PD (Bogerts et al., 1983; Akil and Lewis, 1993; Jin et al., 2019). Whereas the dorsal striatum is primarily supplied by the substantia nigra pars compacta (SNpc), HF/EC mainly receives dopamine from the ventral tegmental area (VTA) (Scatton et al., 1980; Oades and Halliday, 1987; Gasbarri et al., 1997; Rosen et al., 2015) (see Figure 3). Although dopamine depletion is comparable in early PD, the VTA dopamine supply exhibits higher inter-subject variability (Caminiti et al., 2017; Bortz and Grace, 2018).

### Altered somatotopy disrupts body representation

The striatal side of the striato-HF/EC loop with its ontogenetic optimized sensorimotor SBPs undergoes an up to *16-fold* decrease in PD (Cho et al., 2002). This results in SBPs becoming responsible for not one but as many as three or five body parts, becoming fragmented, existing in clusters or isolated cells outside their diminished former clusters (“satellite potentials”) (Cho et al., 2002; Obeso et al., 2008a; Bronfeld and Bar-Gad, 2011; Coffey et al., 2016). There was the argument that these (striatal) “distorted internal body representations… may contribute to bradykinesia, impaired movement scaling, and the strong reliance on visual feedback” (Contreras-Vidal and Gold, 2004, p 505) that seems to be much more attributable to the other side of the striato-HF/EC loop: the GCs with (1) their (distance) scaling properties, (2) their reference to time and velocity (Kropff et al., 2015; Heys and Dombeck, 2018; Carvalho et al., 2020; Heys et al., 2020), and (3) their ligation to external landmarks/cues, with visual cues being the most influential (Maaswinkel and Whishaw, 1999; Hardcastle et al., 2015; Stensola et al., 2015; Chen et al., 2016; Perez-Escobar et al., 2016; Campbell et al., 2018; Keinath et al., 2018; Mosheiff and Burak, 2019; Kinkhabwala et al., 2020).

Compared to the striatal system, the ontogenetically young GC system is highly malleable in its physiological state already (Barry et al., 2007; Langston et al., 2010; Wills et al., 2010; Stensola et al., 2012; Krupic et al., 2015; Latuske et al., 2015; Dunn et al., 2017; Ismakov et al., 2017). Thus, disruptive shifts occur in GCs, with half of grid cells changing to multiple distance and time computations (Kraus et al., 2015), compressing (Raudies and Hasselmo, 2015), skipping (Deshmukh et al., 2010), or even getting lost their grid fields. Even *partial* inactivation may be enough to disturb allocentric computing, necessitating *complete spatial allocentric remapping* (Miao et al., 2015; Rueckemann et al., 2016; Savelli et al., 2017). It is only their robust redundancy and the pooling of all information that allow the complete spatial function of GCs (Reifenstein et al., 2012). The recent discovery of an aperiodic 3D GC pattern in flying bats (Ginosar et al., 2021) implies that there are far more inconsistencies in the real 3D world than in the 2D lattice mazes that shape our current understanding of GCs.

## Hypotheses: dopamine-depleted grid cells evoke Parkinson’s symptoms

We are all too familiar with the debilitating symptoms of PD, yet how they manifest within the framework of the BG-focused box-and-arrow model remains a mystery (Mink, 1996; Brown and Marsden, 1998; Montgomery, 2011). In contrast, when considering the allocentric brain with its grid cells in particular, PD symptoms seem to be self-explanatory, as will be shown below.

### “Conceptual hypometria” as a fundamental symptom in PD

Hypometria may not be the first symptom we bear in mind when discussing PD, but it serves to introduce some key ideas. Just think of clinical signs of hypometria in PD patients, including perceiving distances as shorter (Demirci et al., 1997; Kabasakalian et al., 2013), failing to reach far enough when trying to grasp objects (Klockgether and Dichgans, 1994; Kulkarni et al., 2013; Ryckewaert et al., 2015), underestimating the sizes of objects and openings (Harris et al., 2003; Young et al., 2010; Smith et al., 2011; Laudate et al., 2013), and an impaired ability to perceive large spatial configurations (Barrett et al., 2001; Bernardinis et al., 2018). Therefore, it has been argued that in PD “the sensorimotor apparatus is ‘set smaller’” (Demirci et al., 1997) in line with a constriction of the “perception of *extrapersonal* space” or a *virtual compressed space* (Lee et al., 1998, 2001b; Davidsdottir et al., 2008). This has led to the postulation of not only a virtual egocentric but allocentric hypometria in PD, termed “conceptual hypometria” (Skidmore et al., 2009; Kabasakalian et al., 2013).

Given that GCs represent external space virtually, their dwindling in the context of dopamine depletion (DD) (see above for mEC dopamine receptors; Fallon et al., 1978; Akil and Lewis, 1993; Li et al., 2015) would first and foremost weaken the large spatial allocentric representation because ventral GCs, which represent large spatial scaling, are sparse (Brun et al., 2008; Burgess, 2008; Jeewajee et al., 2008), have a lower signal-to-noise ratio (Bant et al., 2020), and occasionally get lost or switched off (Campbell et al., 2018) in their physiological status already limiting large grid field computations, but favoring smaller ones.

**Figure 4:**
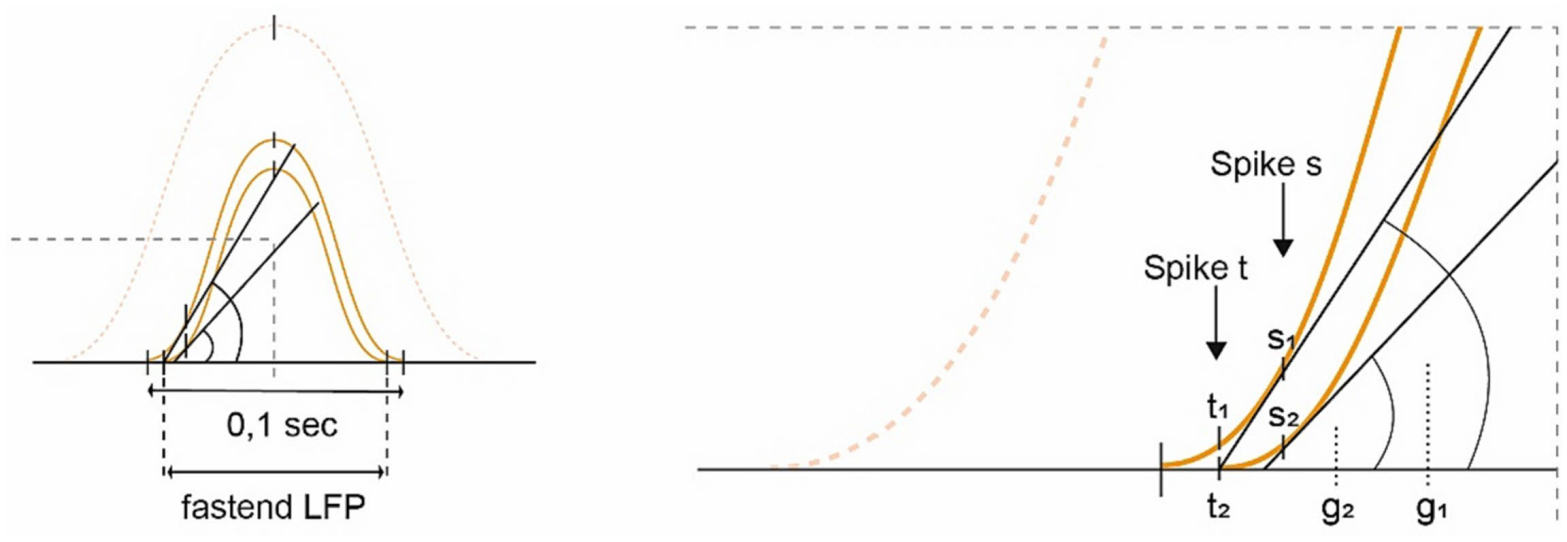
Diagram depicting the idea of “conceptual hypometria.” Building upon Figure 2B, this image demonstrates the effects of losing slower GC modules (dotted sine wave; SW), which increasingly accelerates the remaining ones [shifting from the outer toward the inner small SWs (SW 1 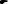 2)]. If the internal firing frequency were to remain constant, the spikes would arrive earlier on the left-hand ascending slope of the fastened and downscaled SW (SW 2), potentially exacerbated by a loss of LFP stability. For spike s, the rise (g2) is smaller in the accelerated SW compared to the original slope (g1). Whereas signal t1 from the stable SW indicates that there is still some distance to cover, t2 of the weakened SW 2 signals “already reached,” substantiating the hypothesis of “conceptual hypometria.”

Furthermore, if the mechanisms of TPP (see above), which normally record the time spent crossing a grid field, are detached from other consistent information or even become inverted in DD, they may signal premature or mistaken spiking. In particular, as the leading GC’s spike yields a clear signature of TPP (Reifenstein et al., 2012), the system might become more volatile with its disinhibition, generating an “already reached” or “closer than” signal and thus restricting the virtual space and impacting the forward planning of velocity (see Figure 4).

All in all, this supports the idea of a downsized allocentric virtual space, in the sense of “conceptual hypometria.” Note that GC function also deteriorates under social conditions due to an increased GC firing rate (Xu et al., 2022), which is not further elaborated on in this paper.

### Bradykinesia

Bradykinesia, one of the primary and most debilitating motor symptoms of PD, is characterized by a marked *slowness* of movement (Marsden, 1989). This symptom has been postulated to arise from the inhibition of the primary motor cortex, a consequence of the overstimulation of the globus pallidus internus (GPi) (Berardelli et al., 2001; Spiegel et al., 2007; Moccia et al., 2014). However, the validity of this model is challenged by the fact that GPi inactivation improves not just bradykinesia, but also its antithesis, L-dopa-induced dyskinesia (LID, see below) (Laitinen et al., 1992; Brown and Marsden, 1998). Consequently, doubt persists regarding this explanation and the model in general (Obeso et al., 2008b; Bologna et al., 2020).

As described above, the concept of time is conveyed to the brain via GCs’ TPP (Harris et al., 2002; Sargolini et al., 2006; Burgess, 2008; Hafting et al., 2008; Climer et al., 2013; Eichenbaum, 2014; Kraus et al., 2015; Kropff et al., 2015; Schlesiger et al., 2015; Hardcastle et al., 2017; Heys and Dombeck, 2018; Tsao et al., 2018; Ye et al., 2018; Jacob et al., 2019; Carvalho et al., 2020; Heys et al., 2020). As an animal moves faster or even accelerates its movement, the internal firing/spiking of grid cells occurs earlier in their physiological state. However, when faced with an errant *virtual* hypometria (see above), the GCs signal as if the animal has moved a greater distance than it actually has what would compute a demand note to slow down or even to stop movement altogether. Bradykinesia could therefore be a secondary effect of “conceptual hypometria” (see above and Figure 4), arguing for limb movements as well as for whole body motion (see below for limitations).

Furthermore, to introduce time into the brain, there are discrete “speed cells” found in GC formation—mainly fast-spiking interneurons, which constitute about 15% of layer II mEC cells (Couey et al., 2013; Buetfering et al., 2014; Kropff et al., 2015). These speed cells are driven externally by neurons from the pedunculopontine nucleus (PPN) and are highly correlated with *future* speed (Rolland et al., 2009; Ryczko et al., 2013; Ryczko and Dubuc, 2017), the PPN again modulating neurons in the MSDB (Justus et al., 2017; Carvalho et al., 2020). The PPN and MSDB are, in turn, both influenced by the LC and dopamine, with the MSDB containing cells that fluctuate as a function of running speed (Zhou et al., 1999; see Figure 3).

In dopamine-deficient conditions, grid and speed cells may lose their anticipatory internal spiking functions. This suggests that grid cells (GCs) have a harder time calculating the upcoming grid field, which is essential for transitioning from the current position to the next, or as Tukker and coworkers write, that changing properties of the grids in altered or novel environments might limit their representational capacity (Tukker et al., 2022), not to mention turbulences caused by dopamine deficiency (DD) of the weakened grid cells themselves, or their decoupling from the striatal egocentric counterpart (see for GC’s malleability above), negatively affecting their accelerating and computational properties promoting bradykinesia.

At the same time, speed cells may become disconnected from future movement (Kropff et al., 2015; Ye et al., 2018). Coupled with the potentially inaccurate activity of the fusimotor system, which acts as a “forward sensory model” too (Dimitriou and Edin, 2010), this can lead to a decrease in movement speed, resulting in bradykinesia.

### Kinesia paradoxa

Kinesia paradoxa (KP) refers to the abrupt shift from bradykinetic to normal velocity, typically brought about by an external cue (Glickstein and Stein, 1991; Distler et al., 2016; Duysens and Nonnekes, 2021). As GCs are thought to generate multiple speed computations (Jeewajee et al., 2008; Kraus et al., 2015; Hinman et al., 2016) for example by skipping theta cycles (Deshmukh et al., 2010; Kraus et al., 2015), see above, the phenomenon of KP can also be comprehended from the GC’s perspective. Skipping from slow to “normal velocity” is probably facilitated by the noradrenergic LC, which allows for “rapid behavioral adaptation to changing *environmental* and (unpredicted) imperatives” (Aston-Jones and Bloom, 1981; Bouret and Sara, 2005), additionally supported by the PPN (Carvalho et al., 2020), which plays a role in escape responses (Caggiano et al., 2018) and drives both grid and speed cells.

### Sequence effect

The sequence effect (SE) is a clinical term denoting the *progressive* shortening of step length observed during repetitive movements influencing handwriting, gait, and speech (Benecke et al., 1987; Agostino et al., 1992; Iansek et al., 2006; Wu et al., 2016). The SE does not improve with dopamine supply (Kang et al., 2010; Lee et al., 2015; Bologna et al., 2020), but tends to occur less frequently in advanced PD (Bologna et al., 2016) and responds positively to visual cues (Iansek et al., 2006; Tinaz et al., 2016). From an allocentric standpoint, this phenomenon could be seen as a form of self-perpetuating hypometria, possibly resulting from the continuous depletion of larger and more susceptible GCs or from a self-reinforcing mechanism focusing on smaller dimensions (this will be further discussed below).

### Festination

Festination, another fascinating parkinsonian paradox, refers to “a progressive shortening of step length, in that case accompanied by a compensatory *increase in cadence*” (Iansek et al., 2006; Nonnekes et al., 2019a), affecting handwriting and speech (Martin et al., 1994; Giladi et al., 2001; Moreau et al., 2007; Nutt et al., 2011) as well. Festination essentially embodies GCs’ characteristics by reducing amplitudes and generating faster speed computations (Jeewajee et al., 2008; Kraus et al., 2015; Hinman et al., 2016). In simpler terms, it equates to pacing in smaller grid fields with compensatory acceleration for the increased cadence (Jeewajee et al., 2008; Kraus et al., 2015; Hinman et al., 2016; Kropff et al., 2021) – the last contradicting being *brady-*kinetic observed such as in freezing episodes and trembling (see below).

### Micrographia

Micrographia is defined as an “obvious reduction in the size of letters” in handwriting. This symptom is observed in up to three quarters of PD patients (Jarzebska, 2006; Wagle Shukla et al., 2012), with about two thirds exhibiting a waning amplitude (Zham et al., 2019) known as progressive micrographia. This pattern is strikingly similar to the SE, as opposed to continuous micrographia (Kinnier Wilson, 1925; Inzelberg et al., 2016; Wu et al., 2016), which appears more hypometric. Importantly, micrographia can be improved with visual cues such as markers or lines (McLennan et al., 1972; Oliveira et al., 1997; Bryant et al., 2010).

An early study on micrographia argued that it “seems to be a compression of words into insufficient space” (McLennan et al., 1972), thus drawing an early connection to the concept of external (virtual hypometric) space. Hypothetically, if the first letters written are related to the external space, the following ones could lose their external spacing for two reasons. Firstly, writing is predominantly an egocentric activity that can overlook its allocentric calibration. Secondly, due to the enlarged SBPs, continuous calibration to a virtual oversized writing gesture occurs, in comparison to an egocentrically represented previous letter (this effect is more pronounced in progressive micrographia).

### Cueing in PD

The utilization of external cues has long been recognized as a powerful tool to improve PD motor symptoms (Martin, 1967; Thaut et al., 1996; Burleigh-Jacobs et al., 1997; Lim et al., 2005; Nieuwboer, 2008; Delval et al., 2014; McCandless et al., 2016; Ginis et al., 2018; Nonnekes et al., 2019b). Flowers argued that “it seems as if the Parkinsonian subject does not seem to ‘know’ where his hand is *in space nor in relation* to other objects, and so must continuously *monitor visually* both his own movement and the *external world* to maintain control” (Flowers, 1976; p. 305). This observation anticipates the underlying egocentric and allocentric structures. When considering cueing and visual guiding in PD, classical PD models offer little insight, but there is evidence pointing to the GC’s significant dependency on external cues (Hardcastle et al., 2015; Chen et al., 2016; Perez-Escobar et al., 2016; Campbell et al., 2018; Mosheiff and Burak, 2019; Dannenberg et al., 2020). This is especially true for visually driven cues (Maaswinkel and Whishaw, 1999; Chen et al., 2013; Kinkhabwala et al., 2020), such as transverse bars, a laser beam, or a companion’s foot, which can enhance movement speed and accuracy, and thereby alleviate freezing of gait (FOG, see below) (Georgiou et al., 1994; Nonnekes et al., 2015; Ginis et al., 2018).

For visual cueing, the mEC not only receives robust input from visuospatial regions (Burwell and Amaral, 1998) but also *direct visual input* from “intrinsic mEC visual cue cells” (Kinkhabwala et al., 2020), object-vector (OV) cells responding to visual contrasts (Killian et al., 2012; Casali et al., 2018; Andersson et al., 2021), and cells responsible for gaze position (Meister and Buffalo, 2018). The latter could be the allocentric counterpart of or be reinforced by visual streaming via the (egocentric) oculomotor loop (Alexander et al., 1986; Fooken and Spering, 2020).

### Freezing of gait

One of the most debilitating symptoms of PD is freezing of gait (FOG), which is characterized by a sudden, transient inability to initiate effective steps, whether when beginning to move (“start hesitation”), turning, or continuing to move (Rahman et al., 2008; Lebold and Almeida, 2010; Vercruysse et al., 2014; Matar et al., 2019). It often occurs when adapting to new forms of locomotion, encountering specific obstacles, or managing a spatial constriction through visual or proprioceptive input (Giladi and Nieuwboer, 2008; Almeida and Lebold, 2010; Nutt et al., 2011; Matar et al., 2019). FOG is notably associated with spatial references, particularly the grid field’s faced floor as occurs when “stepping from one type of surface to another” (Freezing; Parkinson’s Foundation, n.d.).

It has previously been suggested that a “disruption of the representation of external space” contributes to FOG (Lee et al., 2001a; Almeida and Lebold, 2010), pointing again to the role of the allocentric brain. Without sufficient computing the current and the subsequent grid field to facilitate the transition from one to the next—a mechanism seen in place cells (Souza and Tort, 2017)—one might feel “lost in space” or as if “stepping into the void.” FOG is often paradoxically paired with an increased cadence and uncoordinated trembling of the knees (Hausdorff et al., 2003; Schaafsma et al., 2003; Iansek et al., 2006; Jacobs et al., 2009; Nutt et al., 2011), which contradicts the notion of being purely *hypo-or brady*kinetic. These expressions align with the clinical manifestations of FOG (Nakamura et al., 1978; Hausdorff et al., 2003; Plotnik et al., 2005; Okuma, 2006; Plotnik and Hausdorff, 2008; Jacobs et al., 2009; Almeida and Lebold, 2010; Rehman et al., 2019).

Turning—a movement that often triggers freezing (Schaafsma et al., 2003; Spildooren et al., 2010; Mancini et al., 2017; Park et al., 2020)—relies on GCs maintaining consistent interaction with the floor (see GC *phases*) and on conjunctive cells, a fusion of grid and head direction cells, computing turning properties (Sargolini et al., 2006; Keinath, 2016). Internal disturbances of grid and conjunctive cells may disrupt their rotational properties, being “lost in space” or tethering them more closely to environmental borders (see below for the bottleneck phenomenon) (Krupic et al., 2015; Stensola et al., 2015) and thereby precluding turning. Overall, freezing may result from an overload of movement computation in a disturbed allocentric virtual computation (Tukker et al., 2022), particularly during turning when the linearity of grid fields is abandoned.

Freezing of gait typically lasts a matter of seconds or even minutes, aligning with the observation that dramatic GC disruption can even persist for weeks in healthy rats (Savelli et al., 2017). FOG is likely to persist until compensation strategies, such as cueing, are initiated (see above) (Lee et al., 2001a; Schaafsma et al., 2003; Lim et al., 2005; Nieuwboer, 2008; Ginis et al., 2018; Nonnekes et al., 2019b), helping to reconcile and surpass the GC’s ambiguity level (Carpenter and Barry, 2016; Savelli et al., 2017).

Remarkably, there have been reports of patients who were able to ride a bicycle directly out of a FOG episode (while standing on the floor) (Snijders et al., 2011; Kikuchi et al., 2014). This lends support to the idea that FOG is not simply a manifestation of bradykinesia, but that moving away from the disconcerting tessellating floor could resolve the computational deadlock (Freezing; Parkinson’s Foundation, n.d.). Further highlighting the allocentric brain’s responsiveness to cueing (see above), festination can be improved with spatial cues, especially visual ones (Martin et al., 1994; Nonnekes et al., 2019a).

The anatomic structure most commonly associated with FOG is the pedunculopontine nucleus (PPN) (Lewis and Barker, 2009; Virmani et al., 2019; Craig et al., 2020) that—from the allocentric view—drives mEC speed cells (see above and Figure 4), projects strongly via the MSDB to *control the initiation of locomotion* (Fuhrmann et al., 2015), and determines locomotor speed and gait selection (Caggiano et al., 2018; Carvalho et al., 2020).

### The bottleneck phenomenon

Here I discuss a special form of FOG, the bottleneck phenomenon (BNP), which is characterized by a halt or freeze before entering narrow spaces or passageways, or even when navigating close to the edge of a table (Cowie et al., 2012; Gomez-Jordana et al., 2018; Matar et al., 2019). BNP has previously been framed as a perceptual or visuomotor disturbance (Almeida and Lebold, 2010; Cowie et al., 2012; Sidaway et al., 2018).

For an allocentric explanation of BNP, boundary vector cells (BVC)—the ontogenetically oldest allocentric cells (Bicanski and Burgess, 2020)—have to be introduced. BVCs not only provide the intrinsic allocentric framework for native GC metric formation, but also support the continuous stabilization and error correction of GCs (Lever et al., 2009; Bjerknes et al., 2014; Hartley and Lever, 2014; Hardcastle et al., 2015; Stensola et al., 2015; Giocomo, 2016; Stensola and Moser, 2016; Savelli et al., 2017). This is important because when an animal enters a non-familiar environment, it must instantly self-organize a new grid pattern (Hafting et al., 2005; Fuhs and Touretzky, 2006; Barry et al., 2012; Giocomo, 2016; Nadasdy et al., 2017; Stangl et al., 2018) dependent on external landmarks and borders, with the GC system exhibiting the greatest flexibility (Barry et al., 2007; Langston et al., 2010; Wills et al., 2010; Stensola et al., 2012; Krupic et al., 2015; Latuske et al., 2015; Dunn et al., 2017; Ismakov et al., 2017; Keinath et al., 2018). BVC cells respond at specific distances and angles from between one and four boundaries, albeit with gaps between them (Barry et al., 2006; Savelli et al., 2008; Solstad et al., 2008; Lever et al., 2009; Stewart et al., 2014; Bicanski and Burgess, 2020). Especially in the mEC, there are border cells (BC) (Solstad et al., 2008) that respond to proximate boundaries (within “whisker’s range”) that *immediately block* an animal’s path (Hoydal et al., 2019). There are also retrosplenial BCs linked to the mEC that fire “*prospective* to the animal’s next motion” (van Wijngaarden et al., 2020).

**Figure 5:**
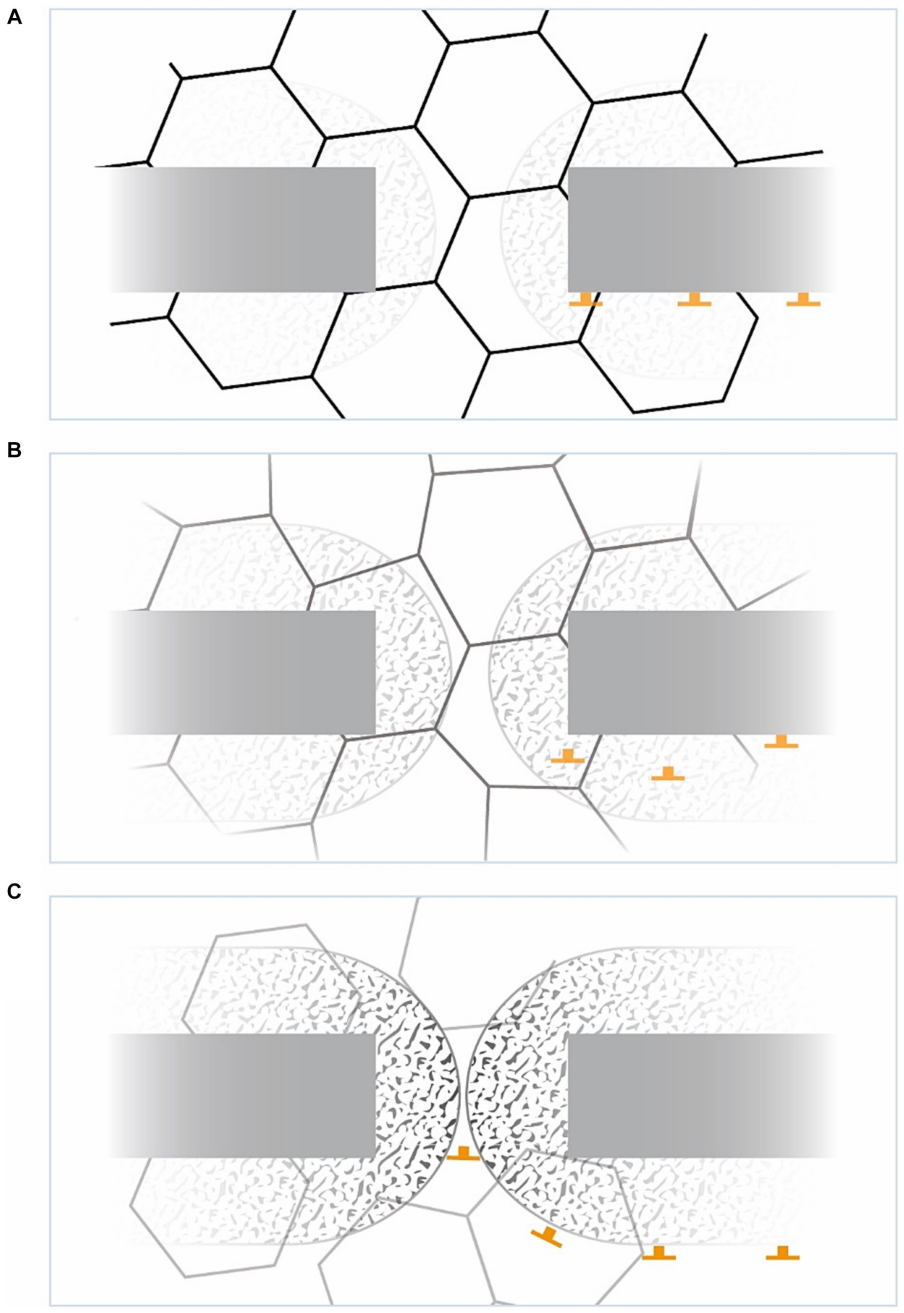
The bottleneck phenomenon from an allocentric view. **(A)** Crossing a doorway, in familiar spaces the area is tessellated with potent grid fields. Grid cells share a border at about 7.5° (Stensola et al., 2015). The area of BVCs (speckled pattern) and border cells (BCs) is shown, the latter with their immediate stop signals (inverted T). **(B)** In weakened GCs, their virtual fields become less pronounced, less structured, but deformed, the BVCs becoming detached from the background and the BCs disinhibited. **(C)** With strengthened BVCs and pushing BCs, movement can be immediately halted virtually, freezing the individuum in space. Note the incremental loss of grid field strength beyond the bottleneck (see text above).

If these cells are disinhibited by deteriorating GCs (Krupic et al., 2015), they virtually generate a stop/freeze signal when standing close to a border. This happens not only in response to borders, but also to doorways enclosed by edges because grid fields are inherently distorted at the edges of the environment (Stensola et al., 2015; Hagglund et al., 2019) bringing BVC and BC to the fore (see Figure 5).

Another potential trigger for the BNP could be the requirement to encode the geometric layout of the subsequent room when leaving the room through a doorway (see Figure 5C). This task may be skipped due to the instability of grid field computations across connected enclosures’ borders (Derdikman et al., 2009; Carpenter et al., 2015; Krupic et al., 2016; He and Brown, 2019). This instability is further compounded by the novelty beyond the bottleneck, which again enlarges and dysregulates grid fields, leading to a brief reduction in spatial stability (Hafting et al., 2005) even in healthy subjects (Figures 5A,B).

### Resting tremor (tremor-at-rest)

Resting tremor (RT), which affects about three-quarters of PD patients, is characterized by an agonist–antagonist motor action in an alert resting position with a frequency of about 4–6 Hz (Milanov, 2001; Hallett, 2012; Zach et al., 2015; Bhatia et al., 2018). Often manifesting a pill-roll component in the distal part of the limb, it is a highly specific sign of idiopathic PD. However, its origin, particularly in relation to the BG, “remains a mystery” (Obeso et al., 2014, p. 524); (Deuschl et al., 2000; Hallett, 2012). Above all, no central pacemakers have been found for PD tremor; instead, there are only cerebral “followers” (Zirh et al., 1998; Hallett, 2014). The independent oscillations of tremulous limbs suggest that individual *body parts* or even single muscles may each have separate tremor generators (O'Suilleabhain and Matsumoto, 1998; Hurtado et al., 2000; Raethjen et al., 2000; Hallett, 2012; Pedrosa et al., 2012).

To explore potential routes, a resting animal or limb needs reliable information about its actual position (Bush et al., 2015; Olafsdottir et al., 2018). If this information is disrupted, such as by the (egocentric) fusimotor disruption, discussed below, or any other kind of computational spatial feedback and if the allocentric brain is unable to store this information during rest due to DD, the GCs’ continuous dynamic error correction system, which again depends on ongoing movement, could lead the spatially related distal limb to seek spatial information through a (searching) movement. This could be exacerbated by unstable egocentric information arising from enlarged striatal SBP, and more so by detached feedback from the hippocampal place cells for GCs’ forward planning during immobility (Olafsdottir et al., 2016, 2017; Zhang and Liu, 2023) or even self-reliant replay (O'Neill et al., 2017).

The antagonistic rhythmicity of tremor could result from striking the virtual edge of the enlarged SBPs, as per the fusimotor “resonance hypothesis” associated with desynchronized long-loop reflexes in PD (Eklund et al., 1982; Zirh et al., 1998). Conversely, it could arise from attaining sufficient positional information and then being deflected from the edges of the associated grid field, possibly in a looser relationship with the LFP. Both peripheral perspectives support the concept of separate body part tremor generation and brain tremor hubs merely acting as “followers.” With the loss of GCs’ stable circular hexagonality, limbs—particularly distal ones responsible for interacting with the proximate, allocentric computed world—may be deflected in their positional scanning, achieving the rotating “pill roll” component.

### Rigidity

Rigidity, a cardinal feature of PD, is present in up to 90% of cases and serves as a primary component of the assessment of dopamine and surgical PD treatment (Andrews and Burke, 1973; Mutch et al., 1986; Powell et al., 2012; Postuma et al., 2015; Powell et al., 2016). Parkinsonian rigidity is characterized by an intrinsically increased muscle tone with clinically uniform resistance to externally imposed joint movement in antagonist muscles throughout the range of motion. Abnormal responses to muscle stretch and long-loop latencies/reflexes have been discussed (Berardelli et al., 1983; Delwaide and Schoenen, 1985; Xia et al., 2011; Pasquereau et al., 2016; Powell et al., 2016).

Alternatively, one could revisit the inconsistent computation of SBP, the “conceptual hypometria” signaling a position beyond the real one, decelerating further movement. That would provide an allocentric explanation for the central proprioceptive disturbances, which are often posited as the actual pathogenesis of PD (Abbruzzese and Berardelli, 2003; Contreras-Vidal and Gold, 2004; Jacobs and Horak, 2006; Fiorio et al., 2007; Rand et al., 2010; Lee et al., 2013). In addition there is also peripheral fusimotor activity of the muscle spindle (Radovanovic et al., 2015), the afferent spinal dorsal horn (Skoog and Noga, 1995; Garraway and Hochman, 2001; Milla-Cruz et al., 2020), and its *efferent* paths along the ventral horn (Yoshida and Tanaka, 1988; Weil-Fugazza and Godefroy, 1993; Barriere et al., 2004; Han et al., 2007; Zhu et al., 2007; Schwarz and Peever, 2011; Clemens et al., 2012; Sharples, 2017; Rivera-Oliver et al., 2019), all of which are disturbed in DD. This apparatus is controlled by the CNS (Ellaway et al., 2015; Macefield and Knellwolf, 2018), with fusimotor activity operating as a “forward sensory model” (Dimitriou and Edin, 2010). Without this—or a determined virtual position to cipher forward motion (Bush et al., 2015; Olafsdottir et al., 2018)—its continuous recalculation could drive the system toward computational overcompensation, resulting in rigidity.

Often, passive turning of an extremity yields cogwheel-like jerks commonly referred to as “cogwheel” rigidity (Ghiglione et al., 2005). This rhythmic unclenching from rigidity may be due to the enlarged and consequently virtually unsubsumable SBPs or their disturbed allocentric link, causing the rigid calibration of tested body parts to break apart and slip into the unknown. The rhythmicity of the “cogwheel” could emerge based on the extent of the virtual egocentric or even ego-allocentric intangible dimensions, or the time delay for allocentric adjustment via the striato-HF/EC loop with a delayed position signal in GCs would prematurely hit the LFP, eliciting a spatial rebound signal and again forcing rigidity. This aligns with the notion of a spatial threshold (ST), the point at which the stretch reflexes and other proprioceptive reflexes activate, modulated by the “corticospinal set” which has been observed to be either hypo-sensitive or even inversely sensitive in PD (Mullick et al., 2013).

### L-dopa induced dyskinesia

“Dyskinesia are involuntary hyperkinetic movements presenting mostly as chorea or choreoathetoid form, but rare ballistic, dystonic or stereotypical variants have been described as well” (McFarthing et al., 2019, p. 449). While these movements develop as a function of disease duration, dopaminergic treatment significantly escalates the probability of their occurrence (Katzenschlager et al., 2008; Nadjar et al., 2009; Nutt et al., 2010; Cilia et al., 2014; Espay et al., 2018; Ryan et al., 2018), largely contingent on plasma L-dopa concentrations. However, LID presents a key paradox in the box-and-arrow model: although therapeutic inactivation of the GPi is thought to trigger dyskinesia, it also alleviates it (Inase et al., 1996; Brown and Marsden, 1998; Desmurget and Turner, 2008; Nambu et al., 2015). LID-associated abnormal neuronal activity has been detected not only in the striatum, but also in the primary somatosensory (Alam et al., 2017) and the primary motor cortex (Halje et al., 2012; Swann et al., 2016).

Reflecting the up to 16-fold enlarged, clustered, fragmented, and at least partially overlapping striatal single body parts (SBP) in PD, along with satellite potentials within remote somatotopic clusters (Cho et al., 2002) and with “neurons that were previously deemed ‘unrelated’ [to movement and that] might now demonstrate movement-related activity” (Bronfeld and Bar-Gad, 2011), LID could signify a disruption of established sensorimotor pathways. Such disruptions disturb their spatial and, therefore, choreographic chronology, promoting chaotic, possibly bizarre movements, deviating from the well-trodden path amplified by a dopamine surplus. In this “unleashed SBP theory,” *hyper*kinesia in LID is no longer interpreted as an acceleration of movement *per se*, but rather as a secondary effect of chaotic or unmanaged somatotopic displacement activity. This theory underscores that “dyskinesia” and “*hyper*kinesia” in PD are not merely the antithesis of being “*hypo*kinetic,” but rather a by-product or the other side of the parkinsonian spatial coin. Therefore, the therapeutic effect of GPi-DBS on LID would not be surprising.

**Figure 6:**
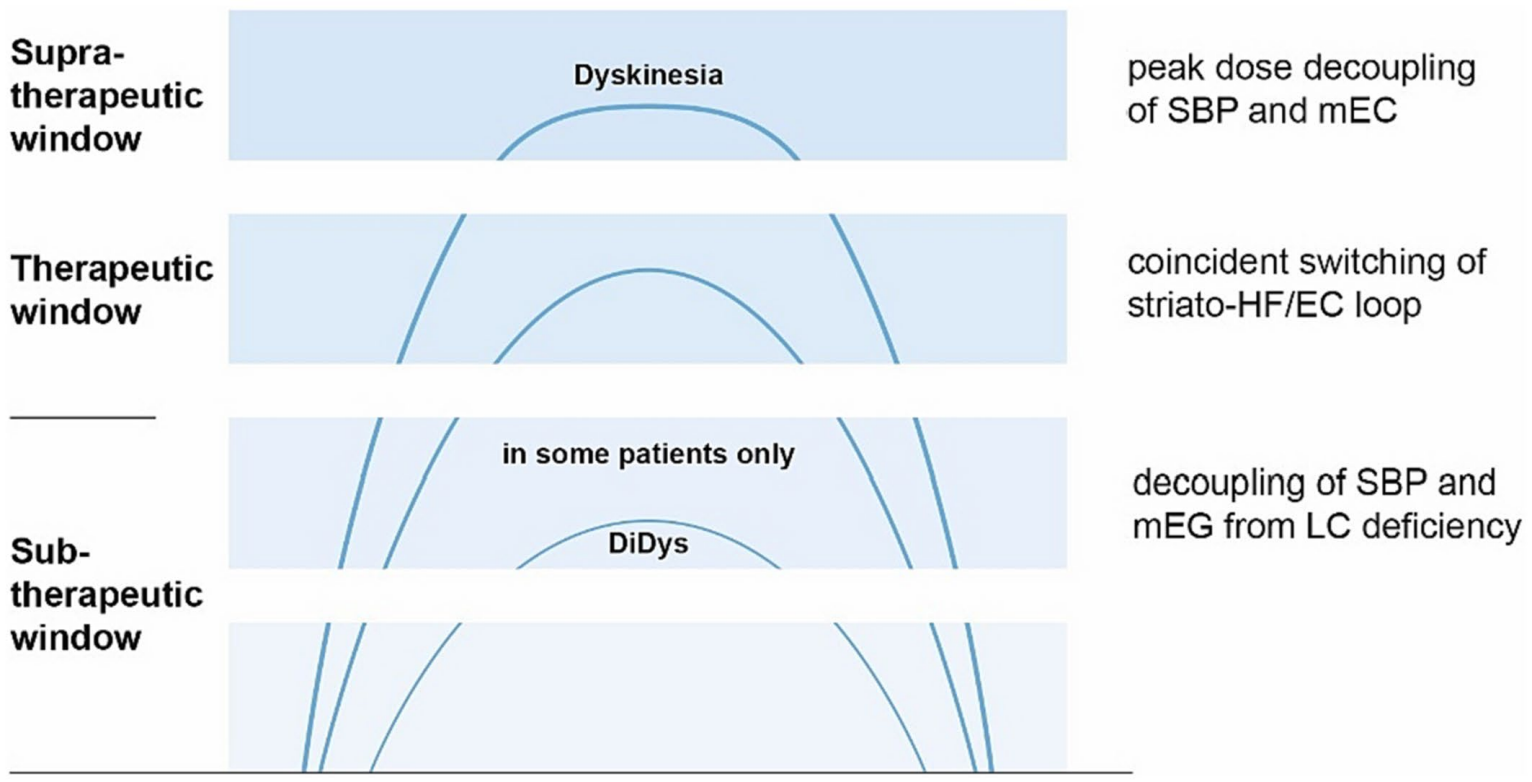
Concept of dyskinesia driven from allocentric central hubs. The *x*-axis represents the time after L-dopa intake, the *y*-axis L-dopa concentration (image based on Espay; Espay et al., 2018). Explanation is shown on the right (top down): Peak-dose dyskinesia arises from the unleashed SBP, decoupled from the ordinarily suppressed mEC (Mayne et al., 2013) from a dopamine surplus. In the therapeutic window, mEC and striatum are balanced by the locus coeruleus (LC) switching the striato-HF/EC loop. In some patients, diphasic dyskinesia (DiDys) occur if LC’s dyskinesia-limiting possibilities (Cedarbaum and Aghajanian, 1977; Miguelez et al., 2011) are inactive, probably due to a lack of dopamine.

Broadening the perspective to the allocentric brain, the SBP could be unleashed from the otherwise stabilizing or balancing mEC due to a dopamine surplus suppressing the mEC (Mayne et al., 2013; Jin et al., 2019) or the LC’s dyskinesia-limiting possibilities (Cedarbaum and Aghajanian, 1977; Miguelez et al., 2011) (see Figure 6). Apart from peak dose dyskinesia, there is lower body predominant diphasic dyskinesia (Manson et al., 2012; Verhagen Metman and Espay, 2017; Espay et al., 2018) occurring just below the therapeutic L-Dopa level. This could meet the criteria for being below the LC’s dyskinesia-limiting possibilities (Miguelez et al., 2011) as well (see Figure 6).

## Limitations

Aside from the hypotheses mentioned, there is, to the best of my knowledge, a dearth of literature discussing the potential role of mEC’s GCs in Parkinson’s disease. Recently, two papers stressed the early involvement of the allocentric brain in PD (Fernandez-Baizan et al., 2020), particularly the mEC (Pieperhoff et al., 2022). Furthermore, evidence suggesting the involvement of EC layer II in parkinsonian symptoms in postencephalitic patients is scant (Hof et al., 1992).

Allocentric “studies have mainly been conducted in simple laboratory settings in which animals explore small, two-dimensional (i.e., flat) arenas” so that “data on the issue of grid cell encoding in 3D are scarce” (Jeffery et al., 2015; Casali et al., 2019; Grieves et al., 2021; Xu et al., 2022). The existence of 3D GCs has been demonstrated in flying bats (Yartsev and Ulanovsky, 2013; Ginosar et al., 2021), and others have identified preliminary 3D grid codes at least in the left human entorhinal cortex (Kim and Maguire, 2019). This study presumes not only the three-dimensionality of GCs and their continuous interactions with striatal SBP, but also its influence on limbs acting in the proximal space liaising the egocentric and allocentric world. However, in the typical allocentric laboratory experiment, foraging—which often concludes with grabbing using the paw or picking up with the snout—demonstrates how the most distant organs complete the link between allocentric guidance and the egocentric world (Heath et al., 2007; Neely et al., 2008). Although allocentric research has already shown hippocampal theta activity accompanying isolated limb movements (Vanderwolf, 1969), there is a gap in the further exploration of this topic.

The hypothesis of translating allocentric whole-body computation to that of the distal body parts involved in goal-directed movements remains largely untested. The extent to which allocentric cells, responsible for completing tasks egocentrically, are present among the many unclassified cells in the mEC remains unknown (Diehl et al., 2017; Miao et al., 2017).

## Conclusion

This paper hypothesizes and illustrates the intriguing association between allocentric properties and PD motor and secondary, spatially related, symptoms in dopamine depletion, with several examples cited throughout. The common thread among these hypotheses is the ambition to surpass the constraints of the box-and-arrow model and the narrow scope of basal ganglia-centric perspectives in PD. Much like other prevailing PD models, these have reached “the point where (their) total rejection, rather than continual attempts at (their) modification, is necessary” (Montgomery, 2011, p. 14). The compelling notion that the allocentric brain influences PD motor symptoms has the potential to substantially shape not only research into movement and movement disorders, but also the broader field of neuroscience.

## Data availability statement

The original contributions presented in the study are included in the article/supplementary material; further inquiries can be directed to the corresponding author.

## Author contributions

AR: Writing – original draft.
